# Herbal melanin modulates PGE2 and IL-6 gastroprotective markers through COX-2 and TLR4 signaling in the gastric cancer cell line AGS

**DOI:** 10.1186/s12906-023-04124-3

**Published:** 2023-09-01

**Authors:** Adila El-Obeid, Yahya Maashi, Rehab AlRoshody, Ghada Alatar, Modhi Aljudayi, Hamad Al-Eidi, Nouf AlGaith, Altaf Husain Khan, Adil Hassib, Sabine Matou-Nasri

**Affiliations:** 1 Department of Biobank, King Abdullah International Medical Research Center (KAIMRC), King Saud Bin Abdulaziz University for Health Sciences (KSAU-HS), Riyadh, Saudi Arabia; 2 Faculty of Pharmacology, Ahfad University for Women, Omdurman, Sudan; 3 Cell and Gene Therapy Group, Medical Genomics Research Department, KAIMRC, KSAU-HS, Riyadh, Saudi Arabia; 4 King Faisal Medical City for Southern Region, Ministry of Health, Abha, Saudi Arabia; 5 Blood and Cancer Research Department, KAIMRC, KSAU-HS, Riyadh, Saudi Arabia; 6 Department of Biostatistics and Bioinformatics, KAIMRC, KSAU-HS, Riyadh, Saudi Arabia; 7 Department of Physics, Faculty of Science, University of Khartoum, Khartoum, Sudan

**Keywords:** Herbal melanin, Gastrointestinal, Gastroprotection, TLR, COX-2, IL-6

## Abstract

We reported a gastric anti-ulcerogenic effect of the *Nigella sativa* (L.)-derived herbal melanin (HM) using rat models. However, the molecular mechanisms underlying this HM gastroprotective effect remain unknown. Cyclooxygenase-2 (COX-2)-catalyzed prostaglandin E2 (PGE2) and toll-like receptor 4 (TLR4)-mediated interleukin-6 (IL-6) production and secretion play major roles in gastric mucosal protection. In the current study, the human gastric carcinoma epithelial cell line AGS was used as a model to investigate the effect of HM on TLR4, COX-2, glycoprotein mucin 4 protein and gene expression using immuno-cyto-fluorescence staining, Western blot technology, and reverse transcription-quantitative polymerase chain reaction (RT-qPCR). Gastroprotective markers PGE2 and IL-6 production and secretion were also assessed using an enzyme-linked immunosorbent assay (ELISA). Bacterial lipopolysaccharides (LPS), well-known inducers of TLR4, COX-2, PGE2 and IL-6 expression, were used as a positive control. We showed that HM upregulated its main receptor TLR4 gene and protein expression in AGS cells. HM increased, in a dose- and time-dependent manner, the secretion of PGE2 and the expression of COX-2 mRNA and protein, which was detected in the nucleus, cytoplasm and predominantly at the intercellular junctions of the AGS cells. In addition, HM enhanced IL-6 production and secretion, and upregulated the *mucin 4* gene expression, the hallmarks of gastroprotection. To check whether HM-induced PGE2 and IL-6 through TLR4 signaling and COX-2 generated, AGS cells were pre-treated with a TLR4 signaling inhibitor TAK242 and the COX-2 inhibitor NS-398. A loss of the stimulatory effects of HM on COX-2, PGE2 and IL-6 production and secretion was observed in TAK242 and NS-398-pre-treated AGS cells, confirming the role of TLR4 signaling and COX-2 generated in the HM gastroprotective effects. In conclusion, our results showed that HM enhances TLR4/COX-2-mediated secretion of gastroprotective markers PGE2 and IL-6, and upregulates mucin 4 gene expression in the human gastric epithelial cell line AGS, which may contribute to the promising beneficial gastroprotective effect of HM for human gastric prevention and treatment.

**Supplementary Information:**

The online version contains supplementary material available at 10.1186/s12906-023-04124-3.

## Introduction

The structural and functional integrity of the intestinal mucosa (*i.e*., epithelium lining the gastrointestinal (GI) tract) are naturally maintained by the balance between aggressive forces and protective mechanisms [1]. The intestinal mucosa layer provides a physical barrier and is the first line of immunological defense against invasion by bacteria, viruses and harmful endogenous macromolecules [1]. Multiple defense mechanisms protect the GI tract, including the mucus gel layer, intercellular tight junctions, mucosal nerves and toll-like receptors (TLRs) [1, 2]. Cyclooxygenase 2 (COX-2) and prostaglandin E2 (PGE2) play a crucial role in mucosal protection [3]. COX-2 and COX-1 proteins are both isoforms of cyclooxygenase (COX) that catalyze PGE2 biosynthesis from arachidonic acid, following its release from the plasma membrane via phospholipase A2 [4]. COX is expressed by the normal gastric mucosa. It contributes to the preservation of the mucosal integrity via PGE2 production [5]. Well known as a mediator of inflammation that regulates tissue regeneration, PGE2 is one of the most important biologically active prostanoids found throughout the GI tract for its physiological functions, including GI secretions and motility [3, 6]. The inhibition of COX-2 and PGE2 causes a decrease in mucus and bicarbonate secretion, reduces mucosal blood flow, and causes vascular injury resulting in mucosal damage [3, 7]. A growing body of experimental and clinical evidence suggests that gastric mucosal damage is mediated through the inhibition of COX-2 and PGE2 by nonsteroidal anti-inflammatory drugs (NSAIDs) [8].

The activation of the COX-2/PGE2 signaling pathway via TLRs has been reported in the GI tract [9, 10]. TLRs are members of a superfamily of transmembrane receptors that recognize pathogen-associated molecular patterns and are a subset of pathogen-recognition receptors. The expression of TLRs has been detected in the GI tract in the epithelial cells, lamina propria, dendritic and Paneth cells [11]. The TLRs expressed on the gastric mucosa are constantly exposed and activated by microbial ligands produced by pathogenic and commensal bacteria. It is acknowledged that the interaction between the gut microbiota and the local TLRs support the maintenance of homeostasis in the GI tract [12]. Various studies demonstrated the expression and activation of TLR4 by bacterial lipopolysaccharides (LPS) in the intestinal mucosa [13, 14]. In addition, the activation of the TLR4/COX-2 and of TLR4/NF-κB signaling pathways induce PGE2 and interleukin (IL)-6 production, respectively, resulting in gastric mucosal protection [15–17].

The IL-6 cytokine, originally identified as a B-cell–stimulating factor 2, is mainly known as an important induced inflammatory modulator, which exerts inhibitory and stimulatory effects on the innate and adaptive immune cells at certain levels of inflammation, in response to infection or tissue injury [17, 18]. IL-6 has pleiotropic activities, which contribute to gastric homeostasis through the regulation of metabolic and regenerative processes, including mucosal repairing [17]. In addition, IL-6 protects the mucosa against ulceration and upregulate mucin 4 expression in cultured gastric cancer cell lines [19, 20]. Numerous studies reported the effects of medicinal natural products, including *Nigella sativa* L. extracts, on antigen-presenting cells stimulating the release of key cytokines such as IL-6, suggesting a beneficial gastroprotective effect of the natural products for the prevention and the treatment of inflammatory diseases, including stomach ulcers [21–25].

Herbal melanin (HM) has been extracted from *Nigella sativa* L. (Black cumin), an annual herbaceous plant in the family Ranunculaceae that widely grows in the Mediterranean countries, Western Asia, Southern Europe, and Middle East [26]. This plant is considered as one of the greatest traditional healing herb and numerous research has been carried out on its medicinal properties [27], such as anticancer [28], antidiabetic [29], antimicrobial [30], hepatoprotective [31], anti-inflammatory and antioxidant [32] agent. HM has been presented as a macromolecule of a heterogeneous polymer mainly composed of 5,6-dihydroxyindole (DHI) and 5,6-dihydroxyindole-2-carboxylic acid (DHICA) [33, 34]. In addition, HM was demonstrated to act via TLRs (i.e., TLR4, its main receptor, and TLR2) leading to NF-κB and p38 MAPK activation, which result in interleukins (*i.e*., IL-8, IL-6, IL-1β) and vascular endothelial growth factor production by human monocytes [35–37]. High HM concentrations exert antiproliferative effects in human monocytic, embryonic kidney and colorectal cancer cell lines through induction of apoptosis [38, 39]. Various studies revealed beneficial effects of melanin on gastric health [40, 41]. We previously reported that HM acts as a strong anti-ulcerogenic agent against gastric ulcers induced in rats [24, 25], suggesting a protective action of HM in the gastrointestinal tract. However, the underlying molecular mechanisms contributing to the gastroprotective effects of HM remain elusive. In the current study, we investigated the gastroprotective effects of HM based on TLR4/COX2 expression, PGE2 and IL-6 secretion using the classical in vitro model for gastric ulcer disease, the gastric carcinoma epithelial cell line AGS.

## Materials and methods

### Reagents

Herbal melanin (HM) was extracted from *Nigella sativa* L. seed coats that were purchased from a local public herbarium in Riyadh (Saudi Arabia). HM was prepared, analyzed and characterized as previously described in [33, 34]. HM was well solubilized in distilled water and the presence of endotoxins in the HM solution was below the detection level following the use of a fluorogenic endotoxin test (Lonza Verviers SPRL, Verviers, Belgium). Culture media and reagents were procured from Gibco® (Thermo Fisher Scientific Inc., Waltham, MA, USA). Lipopolysaccharides (LPS, purified from *E. Coli*) and dimethyl sulfoxide (DMSO) were provided by Sigma-Aldrich Corp (St. Louis, MO). COX-2 specific NS-398 pharmacological inhibitor (#sc-200604) was purchased from Santa Cruz Biotechnology, Inc. (Dallas, TX). TLR4 signaling TAK242 pharmacological inhibitor (#6587/5) was procured from Tocris Bioscience™ (Bristol, UK).

### Cell culture and treatment

The human gastric carcinoma epithelial cell line AGS (#CRL-1739™) was purchased from the American Type Culture Collection (Manassas, VA, USA). The AGS cells were cultured in complete medium composed of Dulbecco's Modified Eagle's Medium (DMEM) supplemented with 10% fetal bovine serum, 2 mM L-glutamine and 1% antibiotics (100 µg/mL streptomycin, 100 IU/mL penicillin). The cells were cultured at 37 °C in a humidified 5% CO_2_-incubator. Reaching 80–90% confluence, the cells were trypsinized and split in a ratio of 1:3 for each passage. Between passage 3 and 8, the cells were used for downstream applications.

The cells were treated with LPS tested at 10 µg/mL, dose reported to exhibit in vivo gastroprotective effects [42], or with various concentrations (5–10-20 µg/mL) of HM at different incubation time periods (4–24-48–72 h).

### Protein sample preparation and Western blot technology

The AGS cells (5 × 10^5^ cells) were seeded in complete medium in 12-well plates. The next day, the cells were incubated in the absence (the Control) or in the presence of either 10 µg/mL LPS or 5–10 and 20 µg/mL of HM, for different incubation time periods (24, 48, and 72 h). The cells were then trypsinized, washed and centrifuged at high-speed. The cell pellet was lysed using the NP40 lysis buffer (Invitrogen, Carlsbad, CA, USA) and the extracted protein samples were estimated using the Invitrogen Qubit™ Protein Assay kit according to the manufacturer’s instructions. The Western blot technology and analysis were done as described in [43] for the detection of COX-2, IL-6, TLR4, phospho-p65 NFκB, and total p65 NF-κB using goat anti-COX-2 antibody (#AF4198, R&D systems, Minneapolis, MN, USA), rabbit polyclonal anti-IL-6 antibody (#TA328217, OriGene Technologies Inc., Rockville, MD, USA), rabbit monoclonal anti-TLR4 antibody (#ab13867, Abcam, Cambridge, UK), rabbit monoclonal phospho-p65 (Ser 529) NF-κB (#44711G, Invitrogen, Thermo Fischer Scientific) and mouse monoclonal anti-p65 NF-κB (#sc-8008, Santa Cruz Biotechnology), respectively. Mouse monoclonal anti-GAPDH (#ab8245, Abcam) and rabbit monoclonal anti-α-Tubulin (#ab13867, Abcam) antibodies were used for the detection of the housekeeping proteins glyceraldehyde 3-phosphate dehydrogenase (GAPDH) and α-Tubulin, used as loading controls. The Western blots were scanned and analyzed using an Odyssey CLx Scanner (LI-COR Biosciences, Lincoln, NE, USA) and ImageJ software (https://imagej.nih.gov/ij/download.html).

### Enzyme-Linked Immunosorbent Assay (ELISA)

The AGS cells (1 × 10^6^) were seeded in complete medium in 12-well plate. The next day, the cells were incubated in the absence (the Control) or in the presence of either 10 µg/mL LPS or 5–10 and 20 µg/mL of HM, for different incubation time periods (4, 24, 48, and 72 h). The levels of PGE2 and IL-6 secreted in the supernatant were determined by ELISA. Human Prostaglandin E2 ELISA kit (#ADI-900–001, Enzo Life Sciences Inc., Farmingdale, NY, USA), R&D systems Prostaglandin E2 Assay Parameter™ Assay kit (#KGE004B) and the human IL-6 Immunoassay Quantikine ELISA kit (#D6050, R&D systems) were used according to the manufacturers’ instructions. The untreated cells, cultured in complete medium, were used as controls. Triplicate readings for each standard, control and sample were recorded as previously described in [33] and measured using a Molecular Devices SpectraMax® Plus 384 microplate reader.

### RNA extraction and reverse transcription-quantitative polymerase chain reaction (RT-qPCR)

Total RNA extraction was carried out from the untreated and treated cells (1 × 10^6^) using the Qiagen RNeasy Mini Kit (Qiagen Inc., Fisher Scientific, Pittsburg, PA, USA) in accordance with the manufacturer’s protocol. The RNA extract was reverse-transcribed to cDNA, and RT-qPCR was performed as previously described in [38]. The primer pair sequences (Invitrogen, Thermo Fisher Scientific) used were 5'-AGATCATCTCTGCCTGAGTATCTT-3' (forward) and 5'-TTCAAATGAGATTGTGGGAAAAT-3' (reverse) for human *COX-2* gene; 5′-GAA GCT GGT GGC TGT GGA-3′ (forward) and 5′-TGA TGT AGA ACC CGC AAG-3′ (reverse) for human *TLR4* gene. The primer pair sequences (Macrogen Inc., Seoul, South Korea) used were 5'-GCTTACTTCAGATGCGATG-3' (forward) and 5'-GTCGAGTTTCATGCTCAGG-3' (reverse) for human *MUC4* gene; 5'-TGCCCAGCTCCTGGC CCGCCGCTT-3' (forward) and 5'-GTGCATCAACACAGGCGCCTCTTC-3' (reverse) for human *COX-1* gene [44]. The gene expression levels were monitored using an Applied Biosystems™ 7500 Real-Time PCR System (Thermo Fisher Scientific, Waltham, MA, USA) and were detected, calculated and normalized to the expression of the housekeeping gene *GAPDH*, as described previously in [38]. The PCR thermocycling conditions for *COX-1* cDNA amplifications and its DNA products separated on 2% agarose gel electrophoresis were processed and visualized as described in [37].

### Immunofluorescence staining

The AGS cells (5 × 10^3^ cells) were seeded on a Nunc® Lab-Tek™ II chambered cover glass. The next day, the cells were exposed to either 10 µg/mL HM or 10 µg/mL LPS at different incubation time periods (24, 48 and 72 h). After the incubation, the cells were rinsed with PBS, then fixed for 30 min at room temperature with 4% formaldehyde diluted in PBS, and the membrane permeabilization was done using 0.1% Triton X-100 in PBS for 10 min, at room temperature. For COX-2 detection, the cells were mixed with either Santa Cruz Biotechnology fluorescein isothiocyanate (FITC)-conjugated mouse IgG1 (#sc-2855, used as a negative control, data not shown) or FITC-conjugated anti-human/mouse COX-2 antibody (#sc-19999). The immunofluorescence staining was captured using LSM780 confocal scanner lasing microscope (Carl Zeiss Microscopy GmbH, Jena, Germany).

### TLR4 signaling and COX-2 generation blockade

To investigate whether HM acts through TLR4 signaling activation and COX-2 generation for the stimulation of IL-6 production/secretion and PGE2 secretion, the AGS cells were treated with TAK242 and NS-398, used as specific pharmacological inhibitors of TLR4 signaling and COX-2, respectively. Briefly, the AGS cells (1 × 10^6^) were seeded in complete medium in a 12-well plate. The next day, the medium was renewed with 1 µM TAK242, 100 µM NS-398 (both were reconstituted in DMSO and concentration was fixed from optimization) and with 1% DMSO (corresponding to the highest pharmacological inhibitor concentration tested), used as a negative control. After 2 h incubation, the cells were exposed to either 10 µg/mL of HM or LPS for a further 72-h incubation, followed by supernatant collection for ELISA and protein extraction for Western blot technology.

### Statistical analysis

All the data are expressed as mean ± standard deviation (SD) based on three independent experiments. A one-way ANOVA followed by post-hoc Tukey test was used for comparison of the two groups. The generalized estimating equations (GENMOD procedure) from statistical analysis system (SAS) software was applied to compare the secreted protein expression level between and within each treatment at the various exposure times. Values of *p* < 0.05 were considered significant.

## Results

### HM upregulated TLR4 and COX-2 expression levels in the human gastric AGS cells

**Fig. 1:**
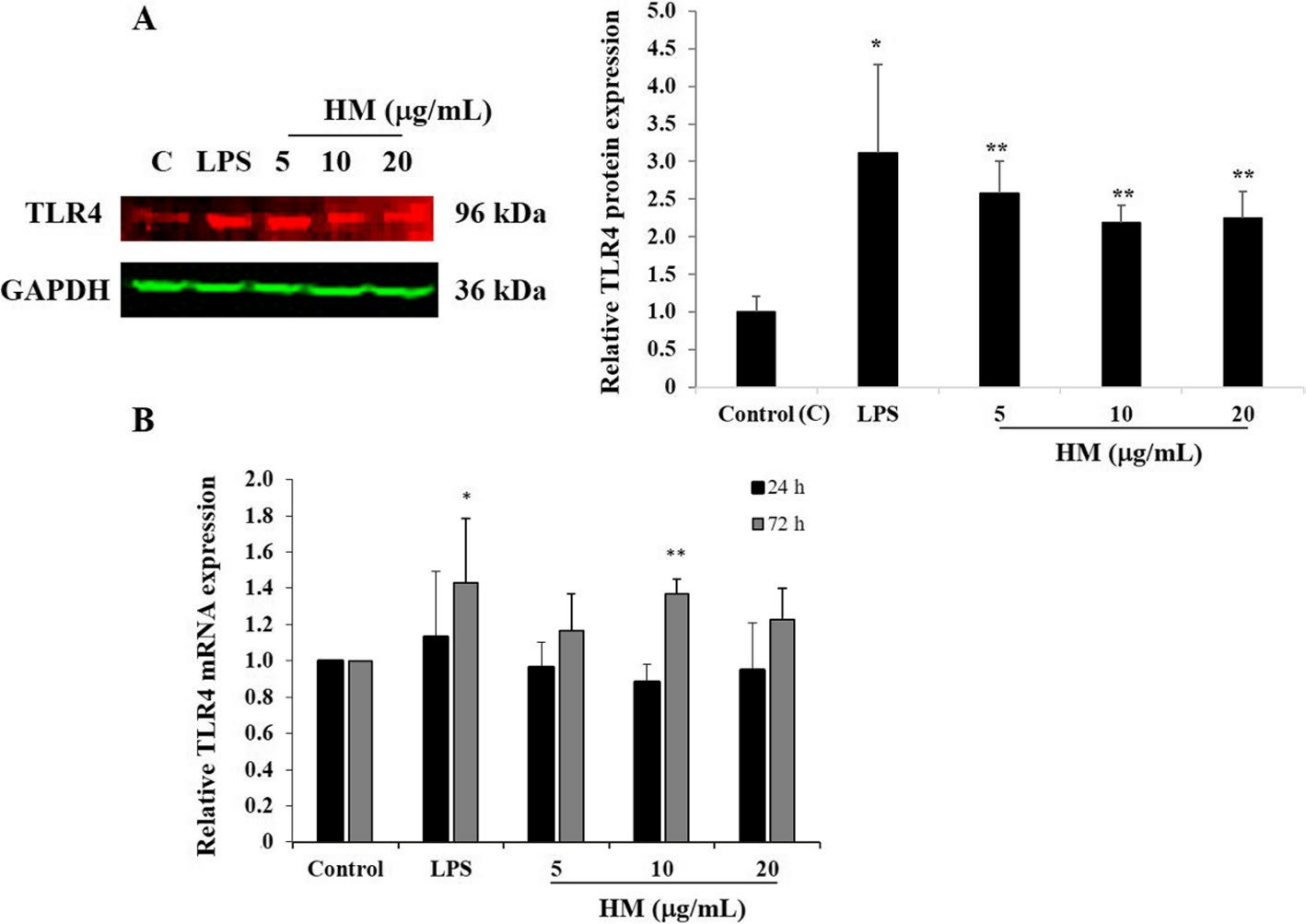
HM upregulated TLR4 protein and gene expression levels in the human gastric cancer cell line AGS. **A** Representative Western blot showing the stimulatory effect of HM and LPS on the TLR4 protein expression after 72 h exposure. GAPDH was used as a loading control. Bar graph indicates the relative expression of TLR4, calculated as a ratio of the expression of GAPDH. **B** Bar graph displaying the relative expression level of the *TLR4* mRNA determined by RT-qPCR analysis in the AGS cells after 24 and 72 h incubation with 10 µg/mL of LPS or HM, compared with the untreated cells, calculated as a ratio of the expression to *GAPDH* mRNA. ^*^p < 0.05 and ^**^p < 0.01 compared with the control, from three independent experiments

TLR4 receptor is known as the main receptor for both LPS and HM, and COX-2 plays a key role in gastric mucosal protection [3, 35]. We monitored the protein and gene expression levels of TLR4 and COX-2 in the AGS cells after 72 h exposure to (5–10-20 µg/mL) HM and (10 µg/mL) LPS, using Western blot technology and RT-qPCR. A significant increase in the TLR4 protein expression was observed in the AGS cells treated with all HM concentrations (2.5-fold, *p* < 0.01) and with LPS (3.11-fold, *p* = 0.037), as compared to the basal TLR4 expression level detected in the untreated cells, the control (Fig. 1A). Monitored at 24 h and 72 h incubation, the HM-induced TLR4 expression was also confirmed at the gene expression level, compared to the untreated cells (Fig. 1B).

**Fig. 2:**
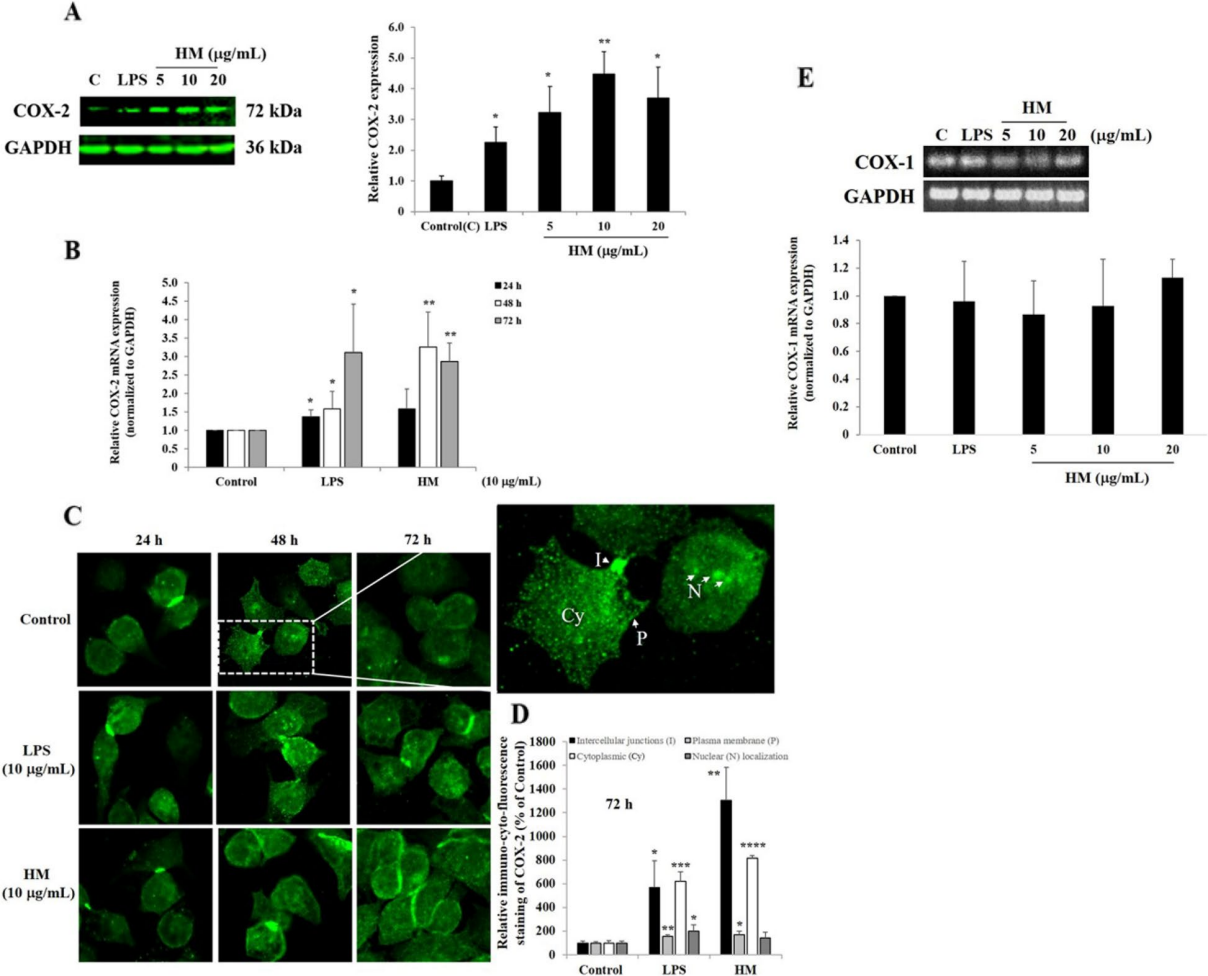
HM increased COX-2 protein and gene expression levels in the gastric AGS cells while COX-1 was not affected. **A** Representative Western blot showing the stimulatory effect of both LPS and HM on COX-2 protein expression in the untreated AGS cells and the cells treated after 72 h incubation. The bar graph shows the relative expression levels of COX-2, calculated as a ratio of the expression to GAPDH. **B** Bar graph showing the relative expression level of *COX-2* mRNA determined by RT-qPCR analysis in the AGS cells after 24, 48 and 72 h incubation with 10 µg/mL of LPS or HM, compared with the untreated cells, calculated as a ratio of the expression to *GAPDH* mRNA. ^*^p < 0.05 and ^**^p < 0.01 compared with the control, from three independent experiments. **C** Representative photomicrographs of the immune-cyto-fluorescence staining of COX-2 expression targeted by monoclonal anti-COX-2 conjugated to FITC, revealing the COX-2 nuclear, cytoplasmic, plasma membrane, and intercellular junctions localizations in the untreated AGS cells and cells exposed to 10 µg/mL of LPS or HM after 24, 48 and 72 h incubation. A higher magnification of an example of the AGS cells depicted in the insert including the arrows pointing to the different COX-2 cellular localizations as “Cy” standing for cytoplasmic, “P” for plasma membrane-bound form, “N” for nuclear, and “I” for intercellular junctions. Scale bar = 5 µm. **D** Bar graph showing the arbitrary quantification of the COX-2 protein expression levels analyzed using ImageJ software (from 6 random fields) at the cellular compartments after 72 h incubation, compared to the COX-2 expression level measured in the untreated cells, the control. **E** Representative gel electrophoresis showing the modulatory effect of both LPS and HM on *COX-1* gene expression in the untreated AGS cells and the cells treated after 72 h incubation. The bar graph shows the relative gene expression levels of *COX-1*, calculated as a ratio of the expression to *GAPDH* mRNA. ^*^p < 0.05, ^**^p < 0.01, ^***^p < 0.001, and ^****^p < 0.0001 compared with the control, from three independent experiments

As depicted in Fig. 2A, compared to the COX-2 basal expression level detected in the untreated AGS cells, the LPS significantly increased (2.25-fold, *p* = 0.015) the COX-2 expression level, and a dose-dependent effect of HM-induced COX-2 protein expression was observed reaching a peak of stimulation (4.47-fold, *p* = 0.0014) at 10 µg/mL of HM (Fig. 2A). The COX-2 gene expression level was monitored in the untreated AGS cells and in the cells treated with either 10 µg/mL LPS or HM at different incubation time periods (24–48-72 h). Compared to the basal *COX-2* expression levels detected in the untreated cells, a slight increase (1.2- and 1.5-fold, *p* < 0.05) in the *COX-2* gene expression levels was induced by the LPS at 24 h and 48 h incubation and a concomitant enhancement (3.0-fold, *p* < 0.05) was observed after 72 h incubation of the cell treatment with LPS (Fig. 2B). After 24 h incubation, HM did not change the *COX-2* gene expression level, but a significant increase in the *COX-2* gene expression level was observed after 48 h (3.2-fold, *p* < 0.01) and 72 h (2.8-fold, *p* < 0.01) of incubation of the AGS cells treated with 10 µg/mL of HM, as compared with the untreated cells (Fig. 2B). Using immunofluorescence staining, representative photomicrographs of the AGS cells incubated with the monoclonal antibody directed against COX-2 conjugated to FITC showed the diverse cellular localizations of COX-2 expressed after 24–48-72 h of cell treatment with 10 µg/mL of LPS or HM. The COX-2 localization was revealed to be nuclear, cytoplasmic, plasma membrane compartments, and at the intercellular junctions (Fig. 2C). After 72 h incubation, significant increases in intercellular junctions-bound COX-2 and cytoplasmic COX-2 expression levels were observed in the AGS cells treated with HM, to a higher extent than the LPS effect (Fig. 2D). Slight significant enhancements of the plasma membrane-bound COX-2 and nuclear COX-2 expression levels were induced by LPS; however, the AGS cell treatment with HM resulted in a significant increase in the plasma membrane-bound COX-2 expression levels, as compared with the cellular localization of COX-2 expression levels determined in the untreated cells, the Control (Fig. 2D). Furthermore, COX-2 is referred to as the inducible isoform; COX-1 is referred to as the constitutive isoform [45]. Unlike COX-2, no modulatory effect of LPS and HM was observed on COX-1 gene expression levels, compared to the basal level detected in the control (Fig. 2E).

### HM enhanced PGE2 secretion from the gastric AGS cells

**Fig. 3:**
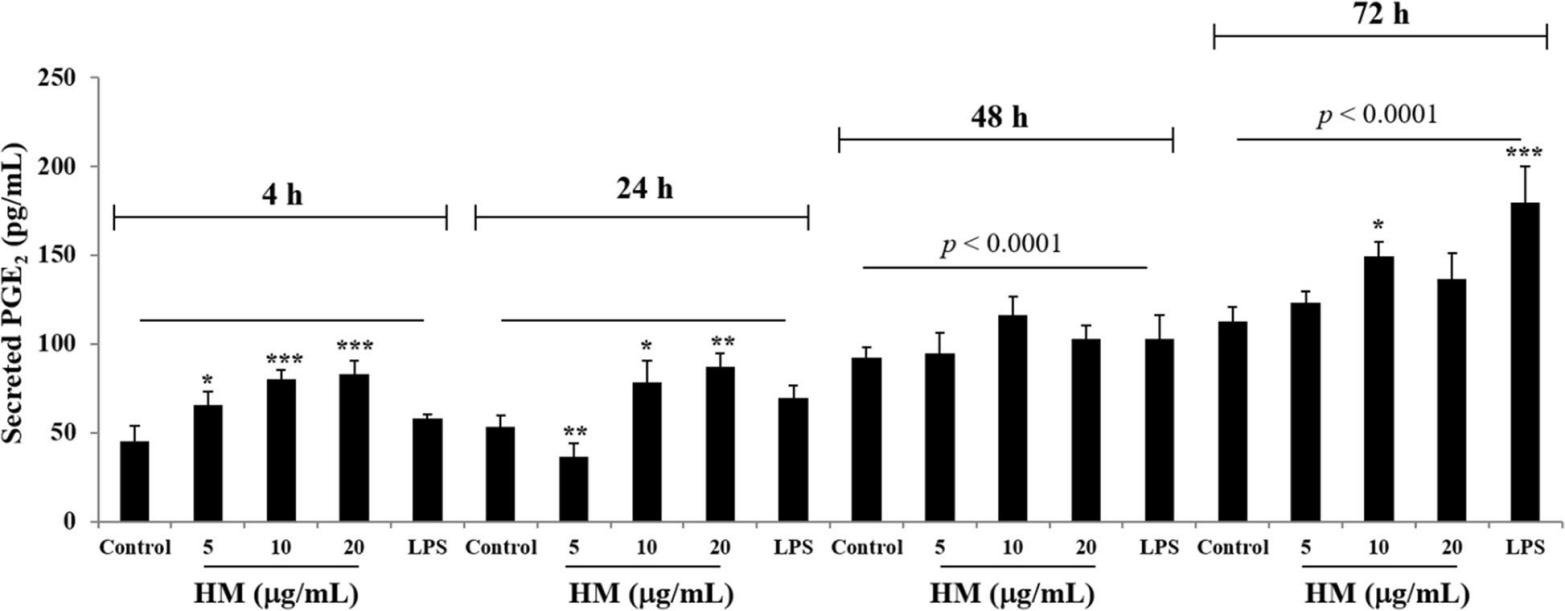
HM enhanced PGE2 secretion released by the gastric AGS cells. Bar graph showing the concentration of the secreted PGE2 determined using the ELISA assay in the collected media of the cultured AGS cells following to 4, 24, 48, and 72 h exposure to 10 µg/mL LPS or 5–10-20 µg/mL HM. ^*^p < 0.05, ^**^p < 0.01, and ^***^p < 0.001 compared with the corresponding control, from three independent experiments

**Fig. 4:**
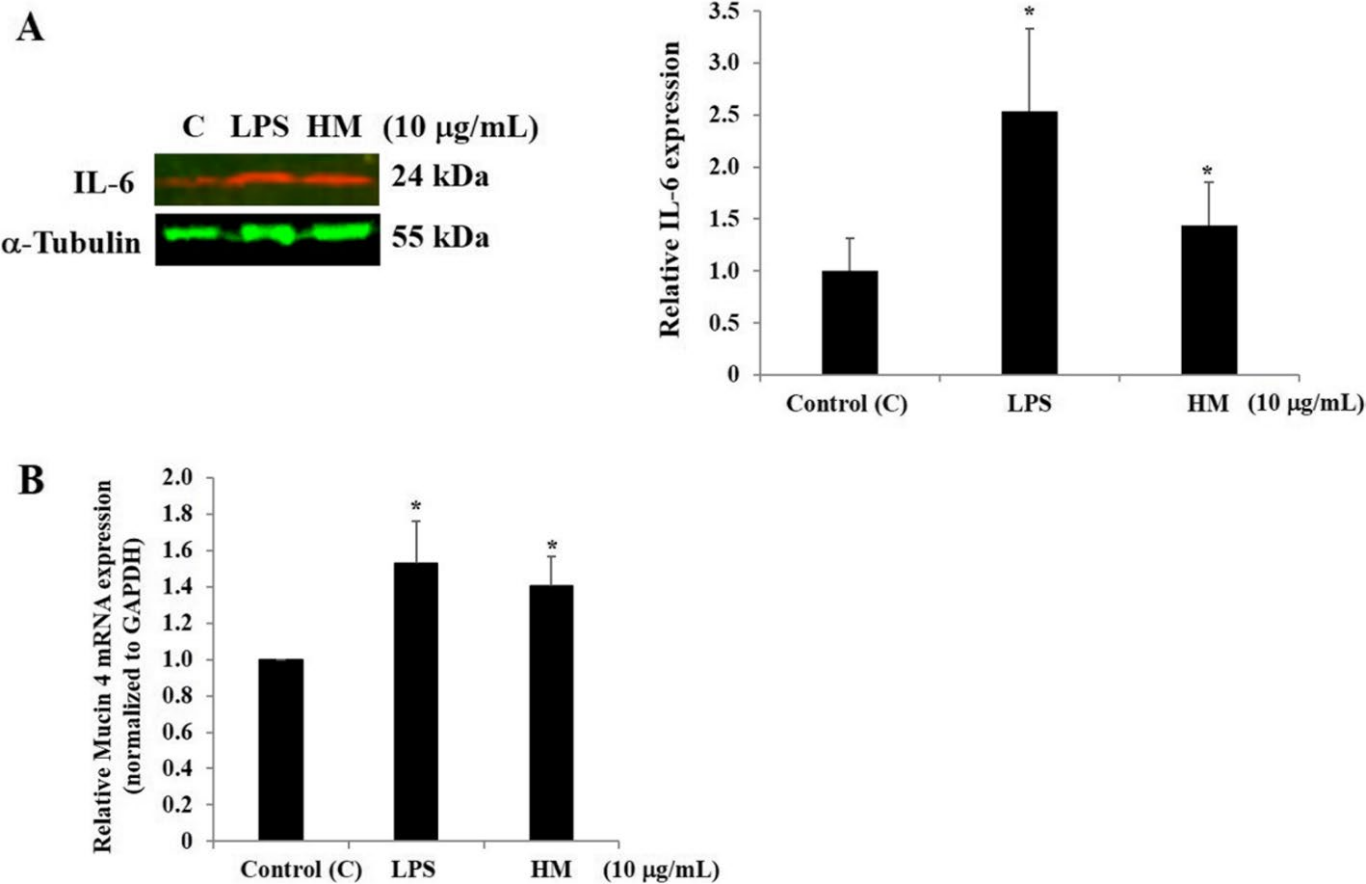
HM upregulated IL-6 and mucin 4 production by the gastric AGS cells. **A** Representative Western blot showing the stimulatory effect of 10 µg/mL of LPS and HM on IL-6 protein expression. α-Tubulin was used as a loading control. Bar graph indicates the relative expression of IL-6, calculated as a ratio of the expression of α-Tubulin. **B** Bar graph showing the relative expression level of *Mucin 4* mRNA determined by RT-qPCR analysis in the AGS cells after 72 h incubation with 10 µg/mL of LPS or HM, compared with the untreated cells, calculated as a ratio of the expression to *GAPDH* mRNA. ^*^p < 0.05 compared with the control, from three independent experiments

With COX-2 described to mediate PGE2 production, the AGS cell culture supernatants were collected after 4–24-48–72 h of treatment with 5–10-20 µg/mL of HM along with 10 µg/mL LPS. After 4 h incubation, a significant increase (1.8-fold, *p* = 0.0006) in secreted PGE2 concentration was observed after the AGS cell exposure to HM. The LPS did not change the secreted PGE2 production as compared with the basal level of the secreted PGE2 detected in the untreated AGS cell culture supernatant collected at 4 h incubation (Fig. 3). A gradual increase of the secreted PGE2 was observed over the incubation time of the AGS cell exposure to different HM concentrations, significant (*p* < 0.0001) at 48 h incubation (2.56-fold increase at 10 µg/mL) and 72 h incubation (3.29-fold increase at 10 µg/mL), compared with the basal level of secreted PGE2 detected in the untreated cells after 4 h incubation (Fig. 3). A concomitant increase (3.96-fold, *p* < 0.0001) of secreted PGE2 was induced by the LPS after 72 h incubation, as compared with the basal level of secreted PGE2 (Fig. 4). Of note, no significant increase of the secreted PGE2 was revealed after the addition of HM (tested at all concentrations) and even after the cell treatment with LPS over 48 h incubation, as compared with the amount of secreted PGE2 released from the untreated cells after 48 h incubation (Fig. 3). However, after 72 h incubation, both the HM (*p* = 0.0324) and the LPS (*p* = 0.0005) tested at 10 µg/mL significantly enhanced the PGE2 secretion by the AGS cells, compared with the amount of secreted PGE2 released from the untreated cells following 72 h incubation (Fig. 3).

### HM upregulated mucin 4 gene expression levels and induced PGE2 and IL-6 production and secretion through TLR4 and COX-2 pathways in the gastric AGS cells

Known as a main immunomodulatory and gastroprotective cytokine produced and secreted via the COX-2/PGE2 pathway [16, 18, 20], IL-6 production was evaluated after the AGS cell treatment with HM along with LPS, using Western blot technology. After 72 h incubation, a concomitant increase in IL-6 protein expression was detected in the HM and LPS-treated cells, compared with the basal IL-6 expression level detected in the untreated cells (Fig. 4A). The gene expression level of mucin 4, a mucosal extracellular matrix protein reported to be upregulated by IL-6 in gastric cancer cell lines [20], was significantly upregulated by HM (1.40-fold, *p* = 0.025) and LPS (1.52-fold, *p* = 0.031), compared with the basal *mucin 4* gene expression level monitored in the untreated cells, the Control (Fig. 4B).

**Fig. 5:**
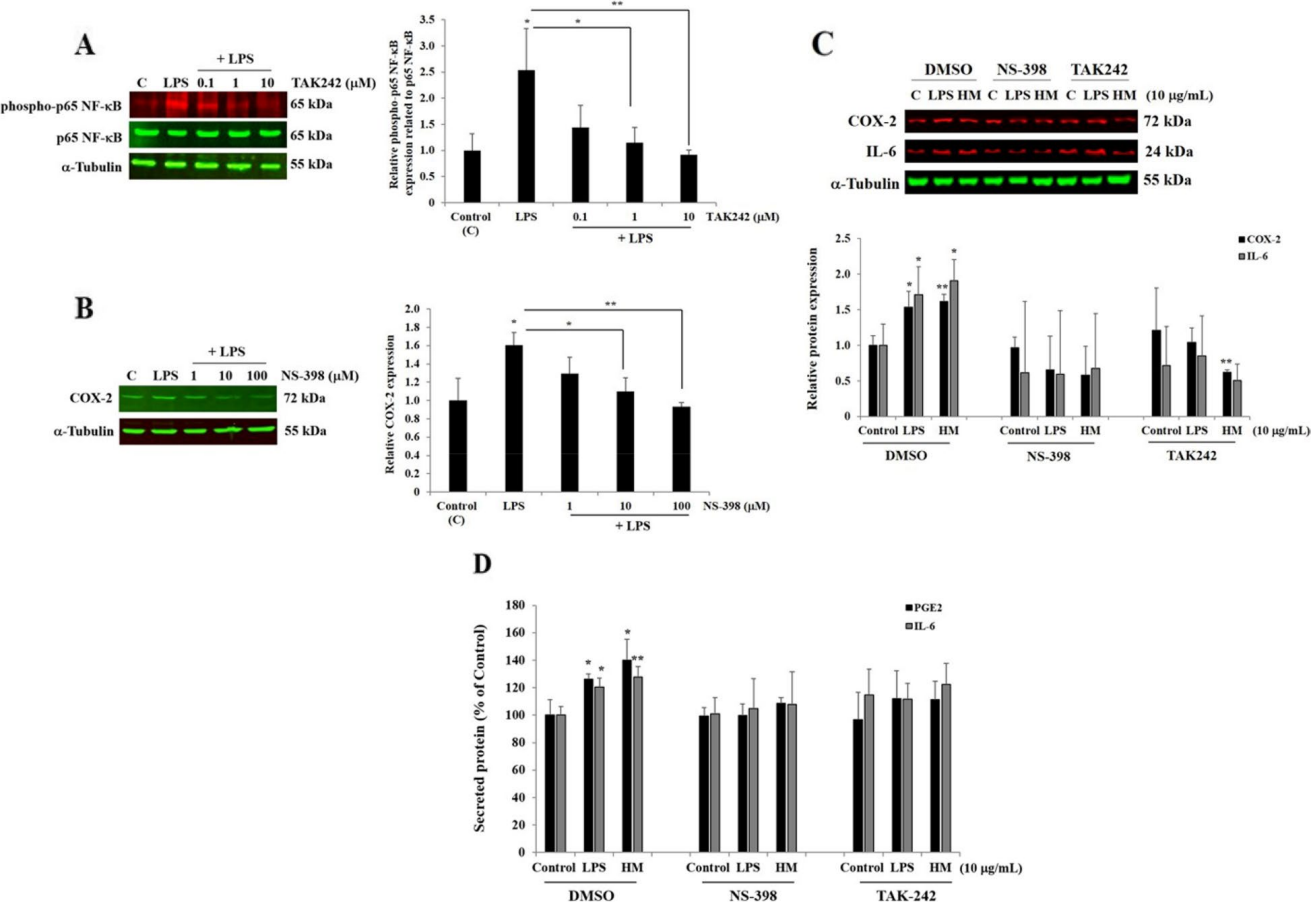
HM-induced IL-6 production and secretion through TLR4 signaling and COX-2 generation by the gastric AGS cells. **A** Representative Western blot showing the optimization of the concentration of the TLR4 signaling pharmacological inhibitor TAK242 blocking TLR4 signaling, based on the loss of phosphorylation of p65 NFκB, its main downstream target. α-Tubulin was used as a loading control. Bar graph indicates the relative expression of phospho-p65 NFκB, calculated as a ratio of the expression of p65 NFκB. **B** Optimization of the concentration of the COX-2 pharmacological inhibitor for the blockade of COX-2 production, based on the decrease of COX-2 expression. Bar graph indicates the relative expression of COX-2, calculated as a ratio of the expression of α-Tubulin. **C** Representative Western blot and bar graph showing and summarizing the impact of the blockade of TLR4 signaling (using TAK242) and of COX-2 inhibitor (NS-398) on LPS- and HM-induced COX-2 and IL-6 production in the AGS cells after 72 h incubation, as compared with LPS- and HM-induced COX-2 and IL-6 production in AGS cells-pretreated with DMSO (solvent used for TAK242 and NS-398 reconstitution). **D** Bar graph showing the impact of the blockade of TLR4 signaling (using TAK242) and of COX-2 inhibitor (NS-398) on the LPS- and HM-induced secreted PGE2 and IL-6 released by AGS cells after 72 h incubation, detected in the conditional media using an ELISA. ^*^p < 0.05, ^**^p < 0.01, and ^***^p < 0.001 compared with the control, from three independent experiments

To explore the main pathways that could be involved in the HM-mediated IL-6 production, the TLR4 signaling pathway and COX-2 generation were blocked, using the pharmacological inhibitor TAK242, a TLR4 signaling pharmacological inhibitor and NS-398, a COX-2 inhibitor. We optimized the optimal use of TAK242 and NS-398 pharmacological inhibitors, tested at various concentrations (0.1–100 µM), based on the phosphorylation of p65-NFκB, the main target of TLR4 signaling, and based on COX-2 generated, respectively. A clear decrease in the phospho-p65 NF-κB expression level (Fig. 5A), as an indicator of TLR4 signaling blockade, and of COX-2 production (Fig. 5B) was obtained after 2 h incubation of the cell pre-treatment with 1 µM TAK242 and 100 µM NS-398 followed by LPS stimulation for 48 h incubation, respectively. Throughout this experimental study with the pharmacological inhibitors, the cell pre-treatment with DMSO, which showed no cytotoxicity, was used as a negative control. The blockade of COX-2 generation using NS-398 decreased as expected the COX-2 protein expression levels in HM and LPS-treated AGS cells, as compared to the HM- and LPS-induced COX-2 protein expression (Fig. 5C). A loss of the LPS-induced COX-2 expression was also observed after the AGS cell pre-treatment with TLR4 signaling inhibitor TAK242 while a significant decrease (0.62-fold, p = 0.009) of HM-induced COX-2 was obtained, compared with the control (Fig. 5C). Regarding the LPS and HM-induced IL-6 protein expression levels detected in the AGS cells, the blockade of COX-2 generation and TLR4 signaling impeded LPS and HM-induced IL-6 production (Fig. 5C). For the quantity of secreted PGE2 and IL-6, determined using specific ELISA kits, a significant increase of both the secreted PGE2 and IL-6 induced by LPS and HM was observed (Fig. 5D). The blockade of COX-2 production and TLR4 signaling attenuated the LPS- and HM-induced PGE2 and IL-6 secretion in the human gastric cancer epithelial cell line AGS (Fig. 5D).

## Discussion

Current treatment strategies for gastric lesions are broadly associated with GI protective and damaging mechanisms. COX-2, PGE2 and IL-6 modulate various functions of the GI tract and have been widely implicated in gastric mucosal protective mechanisms [3, 19]. In recent years, there has been a growing interest in herbal therapies and in the use of natural plant products in traditional medicine to treat gastric lesions. Various studies demonstrated that many plants provide gastroprotection against various ulcerogens and accelerate ulcer healing [46, 47]. *Nigella sativa* (L.) plant, including total extracts or constituents, is one of the traditional medicinal plants that demonstrated various anti-ulcerogenic effects in vivo, though the underlying molecular mechanisms remain elusive [48, 49]. In this study, we investigated the effects of herbal melanin (HM), extracted from the seed coats of the *Nigella sativa* (L.) plant, on COX-2, PGE2 and IL-6 production using the human gastric cancer cell line AGS. We showed that HM enhanced COX-2 expression, *mucin 4* gene expression, and PGE2 and IL-6 secretion, hallmarks of gastroprotection. HM upregulated the gene and protein expression of its main receptor, TLR4. To identify the signaling pathway and investigate the underlying molecular mechanisms involved in HM-induced PGE2 and IL-6 production, the AGS cells were pre-treated with the TLR4 signaling inhibitor TAK242 and COX-2 inhibitor NS-398. An attenuation of HM-induced COX-2, PGE2 and IL-6 was observed in the TAK242 and NS-398-pre-treated AGS cells, indicating the role of TLR4 signaling and COX-2 generated in HM biological effects. We conclude that HM acts through the TLR4/COX-2/PGE2 signaling pathway to induce IL-6 production and HM upregulates the *mucin 4* gene expression in human gastric AGS cells, suggesting a promising beneficial gastroprotective effect of HM for human gastric prevention and treatment. Further in vivo studies are still warranted to determine the gastroprotective effect of HM at the biochemical level, including monitoring the main gastroprotective markers at the tissue and plasma levels.

COX enzymes synthesize PGE2 from arachidonic acid throughout the GI tract and mediate both protective and healing effects [3, 5]. In this study, while HM did not affect gene expression levels of COX-1 known as the constitutive form, the effect of HM on inducible form COX-2 expression in AGS cells was tested using RT-qPCR, Western blot technology and immune-cyto-fluorescence staining. The RT-qPCR data showed that HM increased the expression level of COX-2 in the AGS cells. Similarly, the Western blot analysis showed that the HM augmented COX-2 expression level in a time- and dose-dependent manner. The immunofluorescence staining confirmed these results and revealed for the first time the COX-2 expression in the nucleus, plasma membrane compartments, and predominantly localized in the cytoplasm and at the intercellular junctions of AGS cells upon HM treatment. In addition, we tested PGE2 secretion in the supernatant following HM/AGS treatment. Our ELISA results showed that HM increased PGE2 secretion in a time- and dose-dependent manner. As PGE2 is a direct downstream product of COX-2 [50], the ELISA results confirmed the effect of HM in the COX-2 pathway activation. To further confirm that HM induced the COX-2 signaling pathway, AGS cells were pre-treated with the NS-398 COX-2 pharmacological inhibitor and the results showed a clear reduction of the HM-induced COX-2 and PGE2, which indicate and confirm the direct role of HM in activating the COX-2/PGE2 signaling pathway.

The COX-2/PGE2 signaling pathway acts in combination with other signaling pathways such as the Ras-MAPK and NF-κB pathway and can be activated by different ligands of the TLRs family like TLR4 [15–17]. The role of TLR4 in activating the COX-2/PGE2 pathway in murine macrophages [51], intestinal epithelial cells [10], auditory cells [52], Barrett’s esophagus [53], and in human gastric carcinoma cells [54] has been reported. In the stomach, TLR4 is activated in response to pathogenic invasion, such as *Helicabacter Pylori* LPS, leading to the induction of COX-2 expression and PGE2 production in vivo [55]. The human gastric cell line AGS used in this study was reported to express the TLR4 receptor and the activation of the TLR4 signaling pathway induced by LPS was demonstrated [56]. Previously, we identified HM as a TLR4 ligand and observed a similarity between HM and LPS in TLR4 activation [36]. In the present study, the observed increase in COX-2 expression and PGE2 secretion by AGS after HM treatment was anticipated, similar to the HM effect on the TLR4/COX-2/PGE2 pathway activation. To confirm this assumption and provide evidence of the involvement of TLR4 in the observed results, both the TLR4 protein and mRNA expression levels were monitored. The AGS cells were treated in parallel, with HM at 5–10-20 µg/mL and LPS at 10 µg/mL and Western blot was performed after 72 h incubation. The results showed a clear TLR4 protein upregulation with all the HM concentrations tested or with LPS, as compared with the basal TLR4 expression level detected in the untreated cells. Similarly, HM-induced *TLR4* mRNA expression was confirmed after 24 h and 72 h incubation indicating the definite role of TLR4 in the HM biological modulatory effects. These findings agreed with the reported effects of different TLR4 ligands and COX-2/PGE2 activation on gastric mucosa cells both in vitro and in vivo*.* Fukata and colleagues [10] reported that LPS induces TLR4/COX-2 expression in the human colonic adenocarcinoma epithelial cell lines SW480 and T84 and in the mouse lamina propria macrophages RAW264.7, which results in the upregulation of the mucosal PGE2 detected in the tissue culture supernatants and mouse tissue samples. They proposed that this increased PGE2 expression might be required for mucosal restitution in response to intestinal mucosal injury [10]. Zheng et al. [57] reported that the administration of hyaluronic acid, another TLR4 ligand, activated the TLR4, induced COX-2 and subsequently PGE2, exhibiting protective effects in dextran sodium sulfate-induced colitis in mice. In addition, Chen et al*.* [58] showed that a high-molecular-weight hyaluronic acid protected against induced-gastrointestinal colitis via the activation of TLR4 and COX-2 and PGE2 expression. In these studies, the authors reported the implication of the different TLR4 ligands in the protective mechanism of the GI tract, which suggests a similar gastroprotective role of HM.

The effect of HM on IL-6 secretion and production by AGS cells was evaluated using ELISA and Western blot analysis. Our present results showed that HM treatment enhanced IL-6 secretion and production in the supernatant and cell lysates, respectively. We also showed that both the TLR4 inhibitor and the COX-2 inhibitor attenuated HM-induced IL-6 secretion and production. Our results indicated that HM induced IL-6 via the TLR4/COX-2/PGE2 signaling pathway in the AGS gastric cell line, which agreed with literature demonstrating the production of IL-6 via the activation of TLR4/COX-2/PGE2 pathway both in vitro (i.e., using human coronary artery endothelial cells and tracheal smooth muscle cells) and in vivo (i.e., mouse airways) [59, 60]. However, the blockade of TLR4 signaling and COX-2 generation did not fully suppress HM-induced IL-6 secretion, suggesting the involvement of other HM receptors such as TLR2 signaling, reported to mediate inflammatory cytokine release from human monocytes [37] and keratinocytes [61]. Further in vitro investigation of HM-induced signaling pathways causing IL-6 production and secretion in gastric epithelial cells by targeting TLR2 should provide more insights.

IL-6 is a pleiotropic cytokine acting as a pro-inflammatory and anti-inflammatory cytokine, depending on its local concentration as well as the nature of the target cells [62, 63]. In the GI tract, IL-6 is expressed in the gastric, small intestinal and colonic mucosa epithelial cells and has been recognized as a multifaceted cytokine due to its opposing roles of promoting inflammation and malignancy or protecting and repairing effects [64]. In the stomach, IL-6 plays an important role in inducing cancer cell invasion and maintaining gastric homeostasis at the same time [17, 65]. Studies using mouse models, provided evidence supporting the beneficial roles of IL-6 signaling in protecting intestinal epithelial cells from apoptosis and maintaining the epithelial barrier integrity [66]. However, many studies related IL-6 overexpression to autoimmune diseases and cancer [67]. This controversy in the action of IL-6 has been demonstrated by Gradient et al*.* [68] who reported that IL-6 exerts completely opposite actions on neurons, triggering either neuronal survival after injury or causing neuronal degeneration and cell death in disorders such as Alzheimer's disease. In a previous in vivo study, we demonstrated that HM protected gastric epithelial cells from alcohol-, indomethacin-, aspirin-, stress-, and combined stress with aspirin-induced gastric ulcers [25]. Using human gastric cancer cell lines, Mejías-Luque et al. [20] demonstrated that IL-6 upregulates mucin 4 gene and protein expression levels. In this study, we have shown that HM induced IL-6 in the AGS gastric cell line and increased *mucin 4* gene expression levels, which reinforces the HM gastroprotective potential. A blockade of IL-6 production and secretion would demonstrate whether HM upregulates *mucin 4* gene expression in an IL-6-dependent manner. In spite of the recent advances indicating that IL-6 has multifaceted activities, and perhaps a beneficial role in protecting the GI tract from injuries, we cannot anticipate a protective role of IL-6. Additional in vivo studies are required to elucidate the exact role of HM-induced IL-6 in the stomach.

## Conclusions

The current study is the first to demonstrate the stimulatory effect of the HM extracted from *Nigella sativa* (L.) black seed coats on the TLR4/COX-2 pathway in human gastric epithelial cells, resulting in the enhancement of the secretion of PGE2 and IL-6, accompanied by the upregulation of *mucin 4* gene expression, three key players contributing to gastroprotection. The major limitations of this current study was the use of the classical model for GI disease, the gastric cancer cell line AGS. Therefore, additional studies are warranted to investigate potential HM anti-ulcer activities in vivo through gastroprotective markers contents, assessed at the tissue and plasma levels.

### Supplementary Information

**Additional file 1.**

## Data Availability

The datasets generated and analysed during the current study are available in the In vitro* Gastroprotective effects of HM* repository, doi:10.5061/dryad.ht76hdrj7.

## Abbreviations

cDNA: Complementary deoxyribonucleic acid
COX: Cyclooxygenase
DMEM: Dulbecco’s modified Eagle medium
DMSO: Dimethyl sulfoxide
ELISA: Enzyme-linked immunosorbent assay
FBS: Fetal bovine serum
FITC: Fluorescein isothiocyanate
GAPDH: Glyceraldehyde 3-phosphate dehydrogenase
GI: Gastrointestinal
HM: Herbal melanin
LPS: Lipopolysaccharide
NF-κB: Nuclear factor kappa-light-chain-enhancer of activated B-cells
NSAIDs: Nonsteroidal anti-inflammatory drugs
PBS: Phosphate-buffered saline
PGE2: Prostaglandin E2
RNA: Ribonucleic acid
RT-qPCR: Reverse transcription-quantitative polymerase chain reaction
TLR: Toll-like receptor
