# Supplementary material for: Herbal melanin modulates PGE2 and IL-6 gastroprotective markers through COX-2 and TLR4 signaling in the gastric cancer cell line AGS
Source: BMC Complement Med Ther. 2023 Sep 1;23:305. doi: 10.1186/s12906-023-04124-3 (PMC10474668; doi:10.1186/s12906-023-04124-3)
Supplement: Supplementary file 1 — Additional file 1. [file 12906_2023_4124_MOESM1_ESM.pptx]

## Slide 1
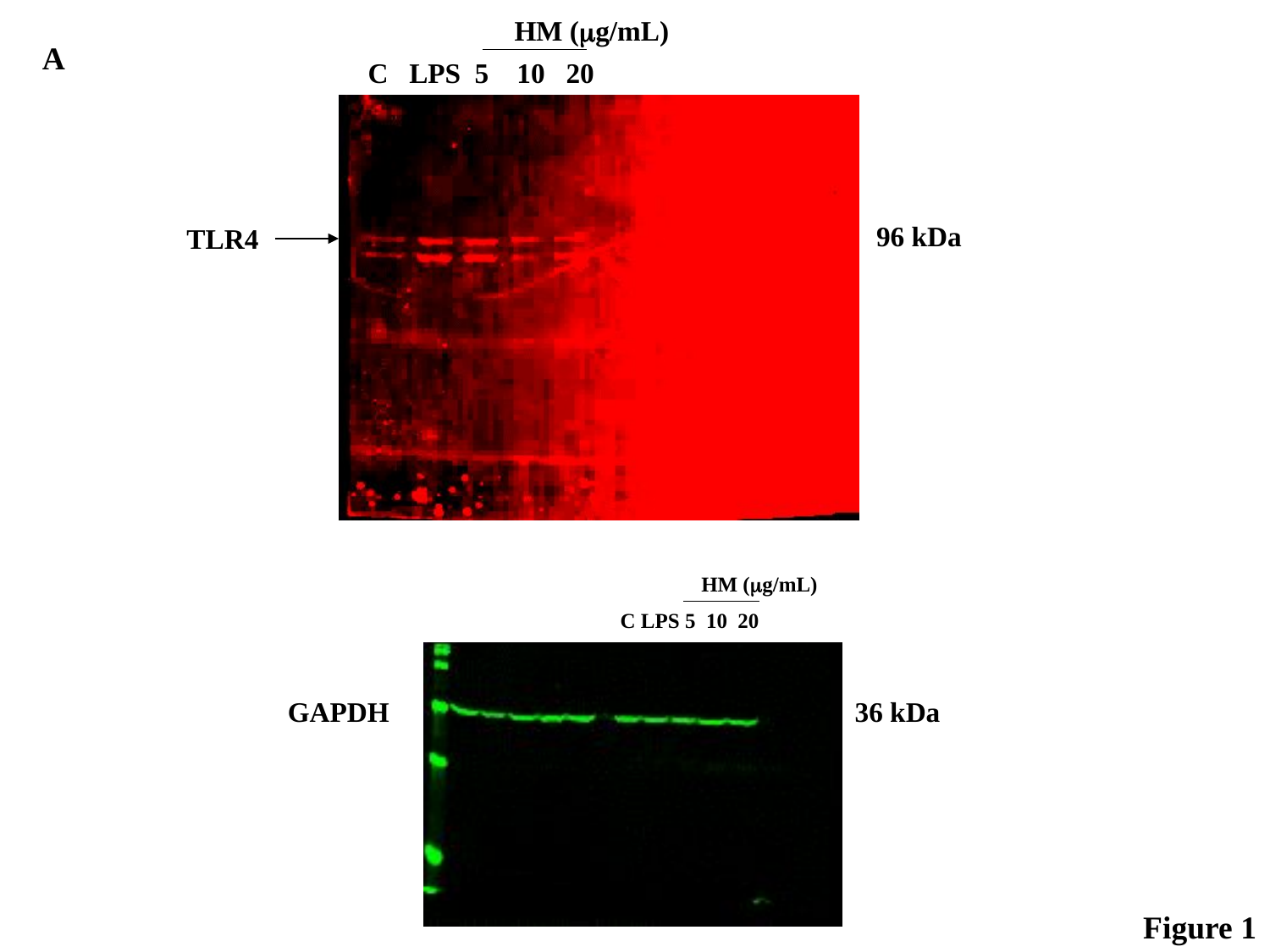

HM (mg/mL)
A
C LPS 5 10 20
96 kDa
TLR4
HM (mg/mL)
C LPS 5 10 20
GAPDH
36 kDa
Figure 1

## Slide 2
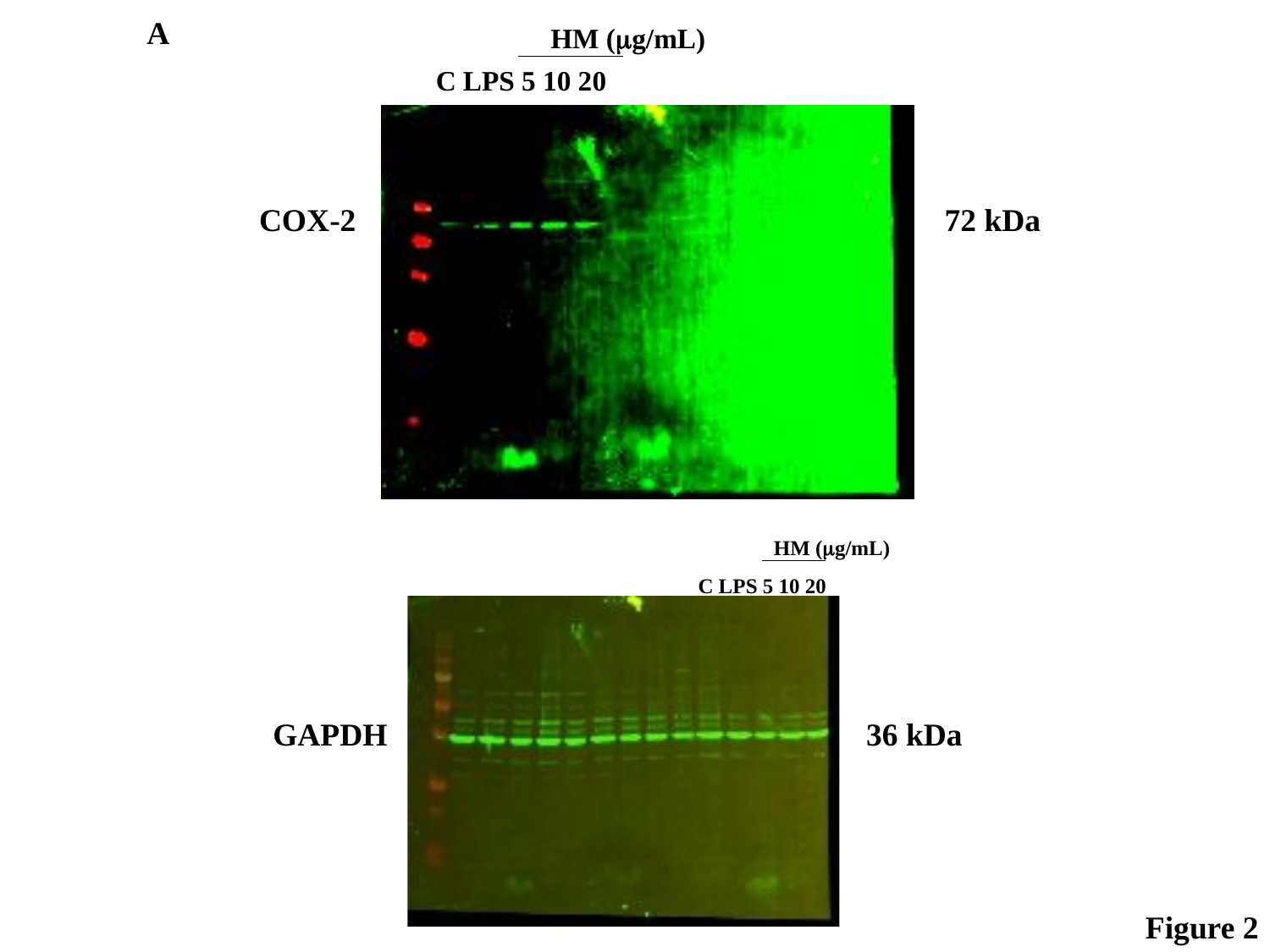

A
HM (mg/mL)
C LPS 5 10 20
COX-2
72 kDa
HM (mg/mL)
C LPS 5 10 20
GAPDH
36 kDa
Figure 2

## Slide 3
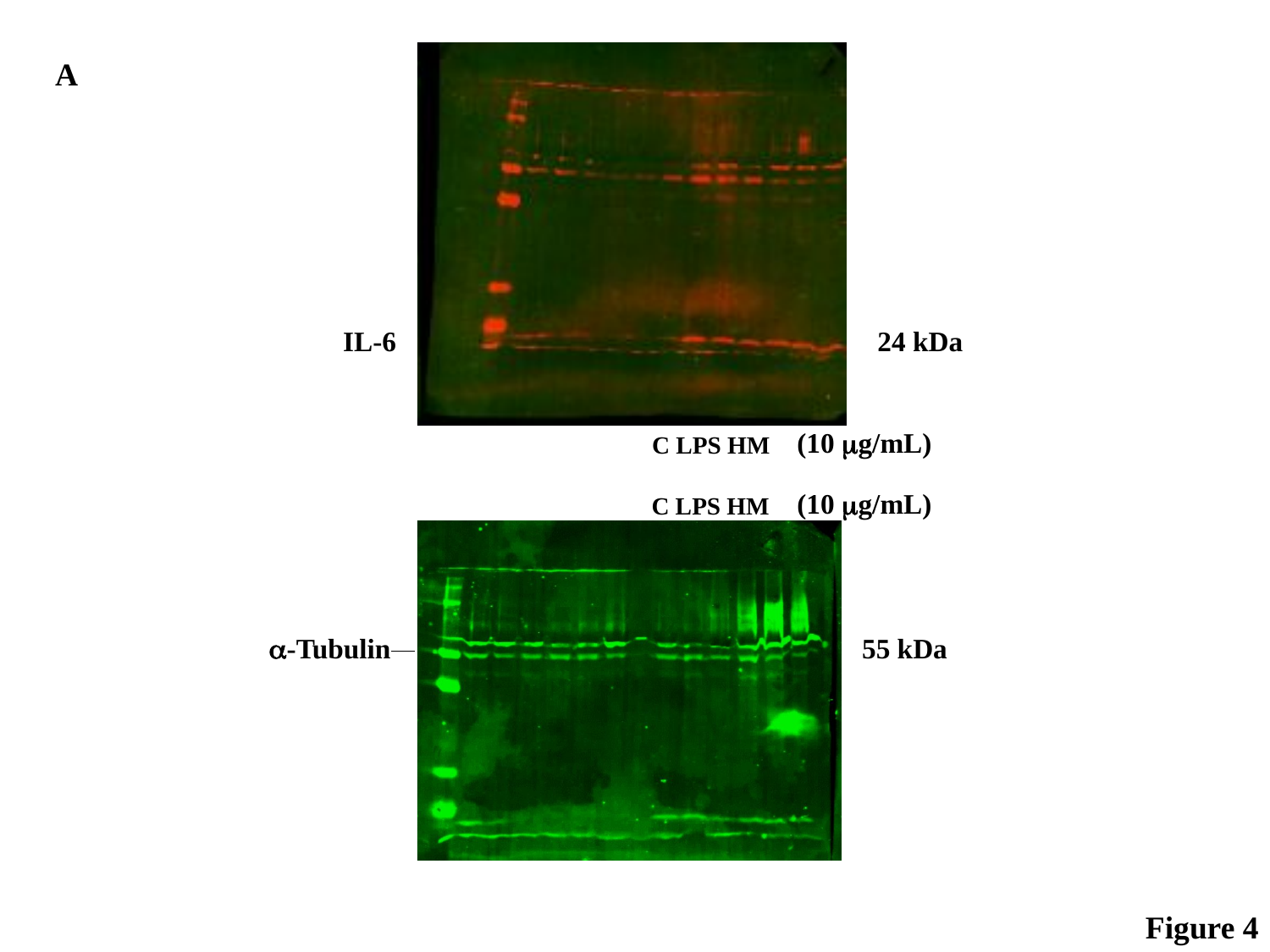

A
IL-6
24 kDa
(10 mg/mL)
 C LPS HM
(10 mg/mL)
 C LPS HM
a-Tubulin
55 kDa
Figure 4

## Slide 4
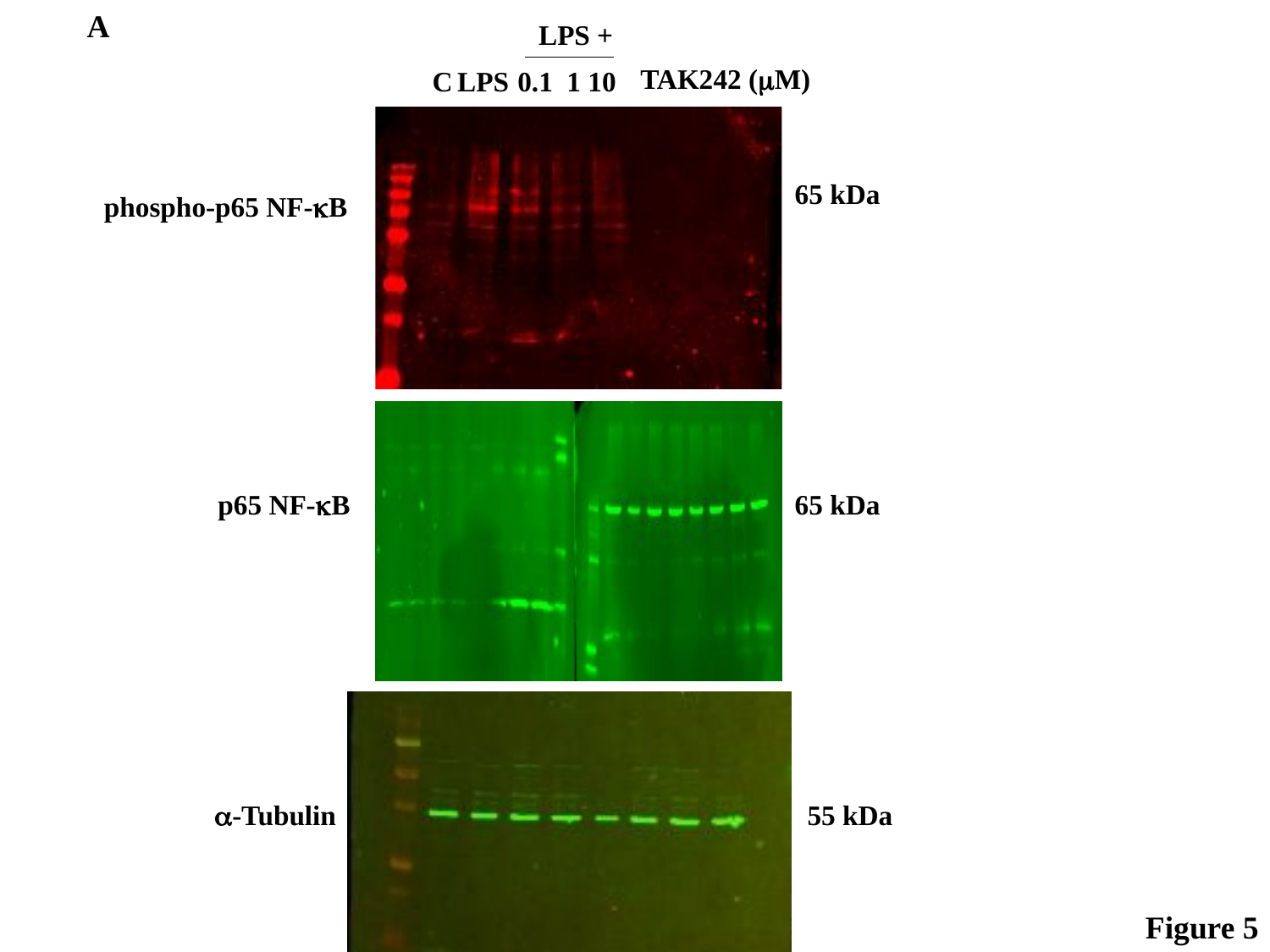

A
LPS +
TAK242 (mM)
C
LPS
 0.1 1 10
65 kDa
phospho-p65 NF-kB
p65 NF-kB
65 kDa
a-Tubulin
55 kDa
Figure 5

## Slide 5
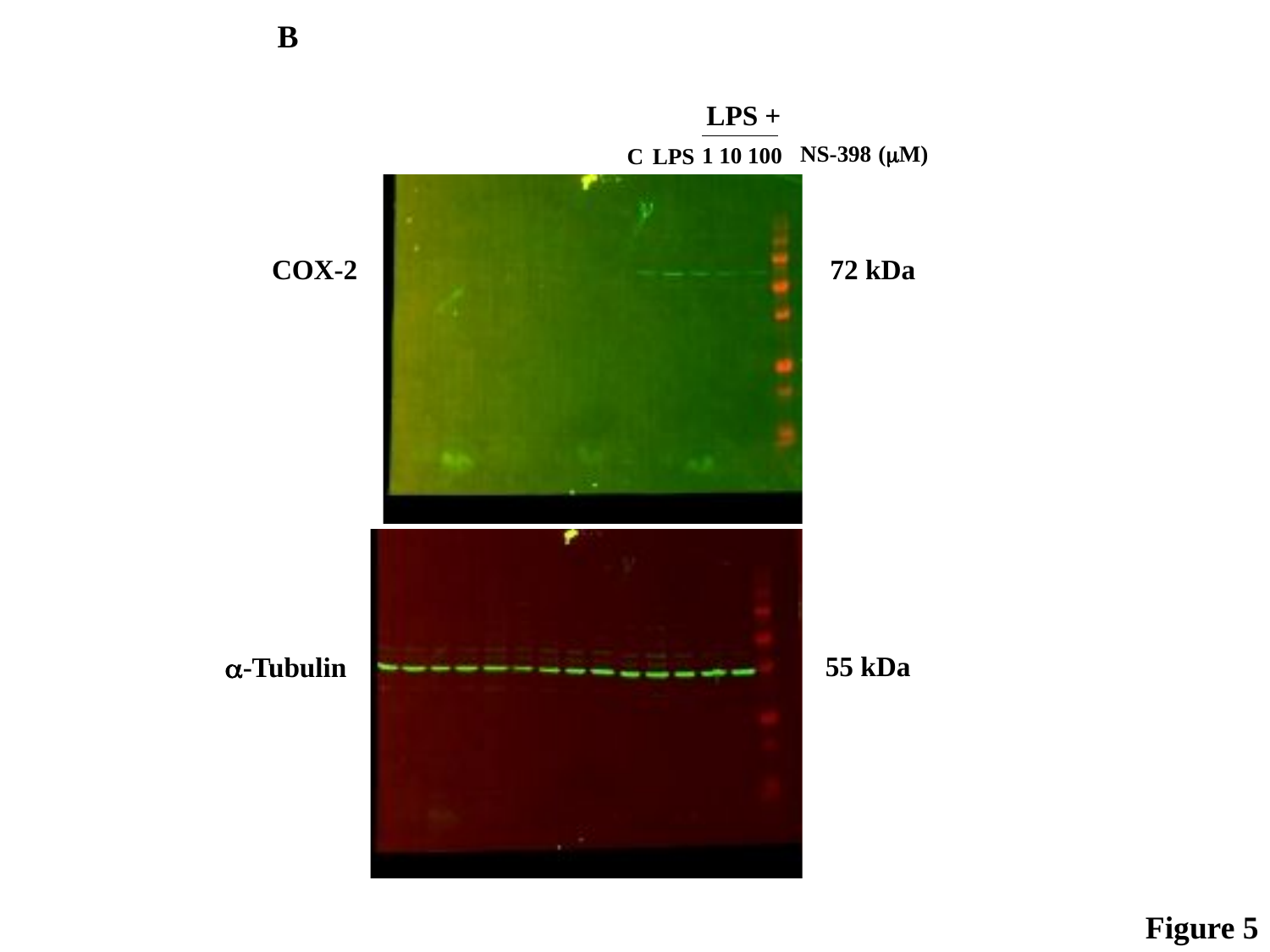

B
LPS +
NS-398 (mM)
 1 10 100
C
LPS
COX-2
72 kDa
55 kDa
a-Tubulin
Figure 5

## Slide 6
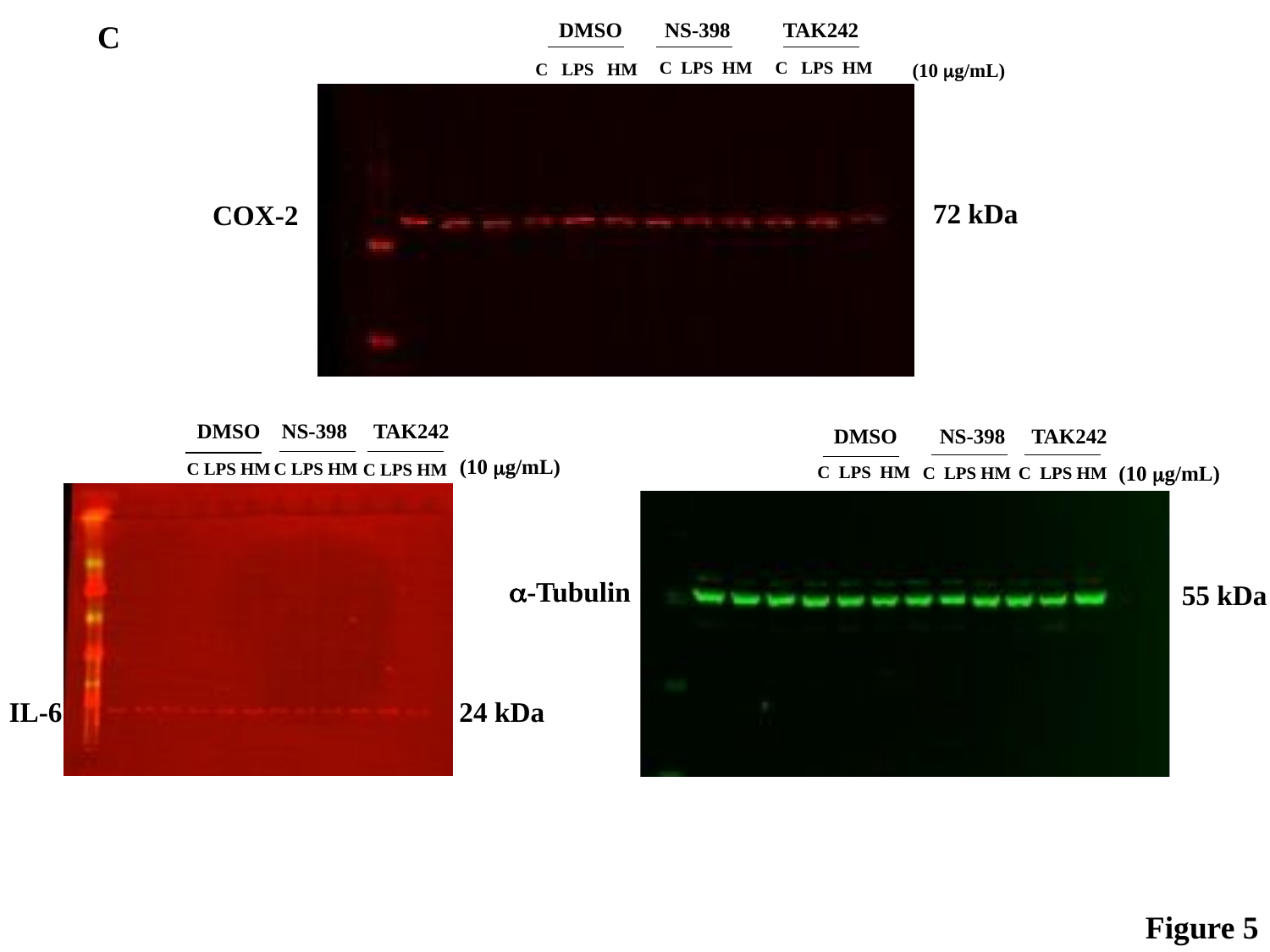

C
DMSO NS-398 TAK242
C LPS HM
C LPS HM
(10 mg/mL)
 C LPS HM
72 kDa
COX-2
DMSO NS-398 TAK242
DMSO NS-398 TAK242
(10 mg/mL)
 C LPS HM
C LPS HM
C LPS HM
(10 mg/mL)
 C LPS HM
C LPS HM
C LPS HM
a-Tubulin
55 kDa
IL-6
24 kDa
Figure 5
